# Hospital and Institutional News

**Published:** 1915-01-30

**Authors:** 


### HOSPITAL AND INSTITUTIONAL NEWS.

### NEW SECRETARY OF HAMPSTEAD GENERAL HOSPITAL.

The vacancy in the secretaryship of the Hampstead General Hospital, created by the sad death of Mr. A. E. Thomas, has been filled by the election of Mr. J. A. Kyle. Up to the year 1911 he was secretary of the Boy Scouts' Association. The eight candidates who appeared before the selection committee were all, with the exception of the successful candidate, well known to hospital officers, and the three selected to appear before the managers were Mr. Richard Allen, secretary of the Royal Hospital, Richmond, Mr. F. P. Carroll, assistant secretary at the Hampstead General Hospital, and Mr. Kyle. A feature of this appointment is that, while the advertisement of the post stated that candidates must have had hospital experience, Mr. Kyle appears to have had no experience of this kind.

### SIDE ISSUES OF THE APPOINTMENT.

The late Mr. A. E. Thomas' successor therefore appears to have been appointed from outside the ranks of hospital staffs, a fact which, while the future should demonstrate its wisdom, is to be mentioned since the phrasing of the advertisement above alluded to may somewhat discourage junior men who are adding up their experience in the hope of improving their chances of promotion. The appointment relieves Mr. Thies of the work as honorary secretary which he has now carried on for six months; and we are glad to see that the hospital committee has adopted our suggestion, and a tablet to the memory of Mr. Thomas is to be placed in the hospital, subscribed for, on as wide a basis as possible, by his friends and all who realise the example which his devotion has set.

### POOR-LAW MEDICAL OFFICERS AND CLINICAL WORK.

Dr. Robson, the medical officer of Alnwick Workhouse, has tendered his resignation to the Board of Guardians because of the increased amount of clerical work required of him under the Poor-Law Institutions Order. At the time the Order was published we drew attention to the large amount of purely clerical work which would devolve upon the medical officer under its provisions, and pointed out the absurdity of allotting such work to him. The medical notes and the directions as to treatment must obviously be kept by the medical officer himself, but he could be saved from much of the purely mechanical part of the work by giving him the right to the whole or part of the time of one of the clerks in the master's office. In most of the large separated infirmaries the medical superintendent has one or more clerks who, acting under his directions, keep the books, fill in certificates and the headings of case-papers and bed-cards, index and file the records, answer the correspondence, get out the material for reports and statistics, and type these returns from shorthand notes or the rough draft. A system of this kind could easily be extended to the smaller institutions, and as the Alnwick Guardians have appointed a committee to consider the matter, we recommend it to their attention as a means of removing Dr. Robson's grievance.

### THE BRITISH HOSPITALS ASSOCIATION AND PAYMENT FOR SOLDIERS.

The secretaries of the voluntary hospitals have been urged by the British Hospitals Association to formulate the views of their committees on the desirability of applying to the War Office for the establishment of a uniform scale in the case of all those institutions where payment is wished for in connection with the treatment of the wounded. In the case of the Red Cross hospitals, except where a private donor has provided hospital and equipment out of his own pocket, the actual sum, whether 2s. or 3s. per bed per day, is based on the certificate of the Deputy Director of Medical Services, and though this sum is smaller than the perhaps 4s. a day accorded to general hospitals, the cases in Red Cross hospitals are, of course, slighter, and, even so, the nature of the cases treated is considered by the Deputy Director before the certificate is issued. Uniformity of such payments is, in principle, always desirable, as in the case of members of Parliament, already decided; and as in the case of dependants of soldiers, on which the decision is being so much delayed. The British Hospitals Association, therefore, with a united opinion behind it, should be able quickly to secure the fulfilment of its wishes in the matter.

### GOVERNMENT PAYMENT FOR THE WOUNDED: AN EDINBURGH VIEW.

We have already drawn attention, in another connection, to the report of the committee appointed at the annual court of Edinburgh Royal Infirmary to consider the managers' report and accounts. Mr. W. A. Tait, C.E., the convener, presented the report to the general meeting of the court of contributors and moved its adoption. After this had been seconded, Sir William Haldane criticised certain passages of the report, particularly one which, he said, referred to the insured class (the working-class) in a patronising manner. Why should they suggest, he asked, it was by favour that the wage-earning classes were admitted? He then moved the deletion of the following passage: "The committee suggest that the possibility of recovering payment from the Government in respect of the wounded should not be lost sight of, particularly in view of the large sums presently being paid by the Government for the provision and maintenance of naval and military hospitals. It would appear that the managers have suspended the normal practice of getting payment from the Admiralty or the War Office, as the case may be. The committee look upon the treatment of such patients as a portion of the cost of the war, and they beg accordingly to recommend that the managers should reconsider their former decision and make representations in the proper quarter upon the matter."

Part of the above passage we have already noted, but Sir William criticised it as being unpatriotic, a going back on their acceptance of the men, and as being not the best way of raising money. His seconder, Mr. G. L. Crole, K.C., took the line that State subvention would mean State control. Mr. Tait reminded the meeting that the managers had for years taken payment for sailors and soldiers getting the benefit of the infirmary. The voting being equal, the Lord Provost gave his casting vote in favour of the amendment, and the amended report was then adopted, after the words "without interfering with the management of the infirmary" had, in deference to Mr. Crole, been added to the paragraph suggesting State payment for what the hospital had done for the insured.

### STAFF SALARIES AND THE WAR.

The facts concerning the work of the year at Leicester Royal Infirmary were of special interest as brought out at the annual quarterly meeting lately held. The first recorded that instructions had been given to the architect for the preparation of plans for an entirely new pathological and clinical laboratory and *post-mortem* room at an estimated cost of £3,500. The building is to be proceeded with with all speed. It is also important to notice the statement of Mr. H. Simpson Gee, the recently elected chairman of the board, who, in moving the adoption of the report, announced that, owing to the war, there has also been large demands made upon the nursing staff, and, in order to maintain the efficiency of this branch of the service, it had been decided to increase the salaries bill of the sisters and nurses by about £500 per annum, an increase of roughly 25 per cent. We record this with peculiar satisfaction, since no hospital manager fully aware of the circumstances fails to realise that in the general under-payment which exists in the ranks of institutional workers the process of improvement has been perhaps most delayed in the nursing department. We recently prophesied that nursing salaries would begin to mend shortly, and Leicester Royal Infirmary's decision is the first instance to prove the truth of this forecast.

### AMERICAN WOMEN'S WAR HOSPITAL.

All the English doctors and nurses with which this hospital was opened at Paignton have now left, and the hospital is staffed, as was the original intention, entirely by Americans, who have, as stated by Dr. Beal, the chief surgeon, come from various parts of America at considerable sacrifice to themselves. The hospital, which at the present time contains 200 patients, has been practically full ever since it started.

### A CHAIRMAN'S POWERS AND A SECRETARY'S RIGHTS.

"At an informal meeting of secretaries," an institutional officer informs us, "the following subject was recently discussed: What are the powers of a hospital chairman with regard to the rights of a secretary? Let us suppose that the chairman thinks it is desirable to conduct a little business at an ordinary meeting without the presence of the duly-appointed secretary, an official who has been voted to the position by the majority of the board. Clearly the chairman must not suggest the temporary retirement of the secretary, unless it is the considered wish of the majority present at any meeting. Should the secretary's salary be under consideration, or should the secretary have been guilty of any misconduct, we can easily understand that it were better he should not be in attendance when a discussion takes place, but on any other occasion no chairman ought to ask a competent and trusted secretary to leave a meeting; it would be a slur upon the latter's professional status. Any job that is engineered at a public institution is a discredit to a secretary as much as it is to a board, or a committee, and if a secretary is not allowed a free voice in the matter, he would be entitled to a clear point of the fact being made in the annual report, or in the chairman's next remarks before the governors." It appears that a number of divergent views were expressed. What are our readers' opinions?

### FROM LAUNDRY TO X-RAY DEPARTMENT.

The present war has given many remarkable instances of the transformation of buildings to hospital purposes, and we can note at the same time, though not primarily for war purposes, the installation of an *x*-ray department in what was the old laundry at Ashton-under-Lyne District Infirmary. Dr. J. W. Morison is in charge of the department, and has been giving demonstrations of the new apparatus to members of the Infirmary Workpeople's Committee on Saturday afternoons. A topical interest to these demonstrations was given by the display of negatives showing the movements of German bullets and shrapnel after entering the bodies of the wounded. It was noteworthy that at the conclusion of the first demonstration, when thanks were tendered by Mr. G. Peagram, president, and Mr. James Clough, secretary of the Workpeople's Committee, it was remarked that the lecture and demonstration "would give them heart to appeal for strong support for the institution by the workpeople of the district." We give this as a good and intelligent means of arousing a proper pride and awakened interest in the hospital, and commend it to such institutions as the Hertford County Hospital, for instance, as typical of the proper and statesmanlike way in which the public may be encouraged to become intelligent supporters of hospital work.

### THE CHADWICK GOLD MEDALLISTS.

We published last week the substance of Dr. F. M. Sandwith's first lecture on "War and Disease," dealing with Continental wars of the nineteenth century, at which the approaching award of the Chadwick medals and grants of £50 each "to such naval and military medical officers who shall have most distinguished themselves in promoting the health of the men of the respective Services" was announced. The award was made at the second lecture, after we had gone to press on Friday last week, when Sir William Collins, Chairman of the Chadwick Trustees, presented the medals and award to Colonel Heaton Horrocks, R.A.M.C., and to Fleet-Surgeon Cleveland Munday. The chair was taken by Sir Frederick Treves, and the prize-winners were nominated by Sir Alfred Keogh, R.A.M.C., Director-General, and Sir Arthur May, Director-General of the Navy Medical Service. Before the delivery of the second lecture, which we report fully on another page, the presentation took place, when Sir Arthur May thanked the trustees for the honour they had conferred on the Navy. When he entered the Navy in 1878, he remarked, the rate per 1,000 of men daily sick was 47 during the Russo-Turkish War: to-day it was only 25. The invalid rate has fallen from 35 to 15 during the same period. The death rate was 6 per 1,000, and is now 2, thanks to sanitation and the education of the men in personal hygiene. The hygiene of ships, he added, used to be sadly deficient, but there had been a gradual fighting of the constructors of the ships, who never went to sea themselves, for better vessels. Fleet-Surgeon Munday then received his prize.

### THE CHADWICK MILITARY MEDALLIST.

The Military Medallist, Colonel Horrocks, was then introduced by Sir Alfred Keogh, who enlarged on the importance of the health of an army in preserving its fighting strength. Colonel Horrocks, he remarked, was the officer most responsible for the very satisfactory state of the health of the Army now in France. His work had extended over a number of years, and it was well known that the abolition of Malta fever from Malta was due to him. Formerly some 600 or 700 men in that island were stricken with that disease in a year. Apart from the sickness and suffering, it cost in hospitalisation alone £15,000 per annum. To-day there was no Malta fever. Colonel Horrocks had also carried on, quietly but persistently, war for the improvement of camp and barrack sanitation. It is also interesting to note that Colonel Horrocks has been appointed Honorary Physician to the King.

### A POOR-LAW INSPECTOR ON THE EFFECT OF THE WAR.

Addressing the North Bierley Board of Guardians the other day, Mr. P. H. Bagenal, the Poor-Law Inspector for the district, dealt with the effect of the war on Poor-Law institutions. After remarking that an inspector was told by his critics that if he did not do something for his salary he was not worth it, he said that they had never grudged the money he had asked them to spend. There were always new developments in Poor-Law administration, and he felt very strongly that one of the results of the present war would be that Poor-Law hospitals would be very much in request, and that their existing accommodation would be found insufficient. No classification was possible at present, and they were using a certain number of beds in a way they would not have to do if they had a larger number of severe cases. In two large wards on the female side that day not a single case was in bed. Constant attendance, but not necessarily skilled nursing nor expensive buildings, was required. He looked forward to a time when some of their cases might be so dealt with as to make room for others of an acute kind, such as might be expected after the war. Their present imbecile wards were not really fit for that class of case, and if the block could be gutted and made into really good wards it might enable them to accommodate and classify the kind of case he had mentioned. The imbeciles could then either be boarded out or an arrangement made to join with some other Union. The good many children in their wards should be dealt with quite apart. There was a large ward at their disposal, but they had infectious cases to deal with. He recognised the board as one of the first to accede to the wish of the Local Government Board to get the children out of the "House" as far as possible. Mr. J. A. Law, chairman of the infirmary committee, then said he had been wondering whether they might not think it best to build a children's hospital, the effect of which would be to leave considerable space available, and as regards the imbeciles, they might set up a small colony if they could get the sanction of the Local Government Board. The need for centralisation between the Unions and classification within the institutions has never been better exemplified, and if the war creates a necessity for hastening the consummation of these reforms it will have produced one solid change for the good in the Poor-Law Service.

### AN OFFICIAL EXPERIMENT WITH CLINICAL ASSISTANTS.

The Local Government Board have notified the Lambeth Board of Guardians that they approve of the appointment of clinical assistants to the medical staff at the infirmary for an experimental period of six months. As previously reported in the columns of The Hospital for December 5, 1914, page 214, the Guardians had considerable difficulty in obtaining the services of qualified medical men to fill the vacancies caused by the war, and rather than engage an outside practitioner to assist in the work at the infirmary, they resorted to the engagement of clinical assistants, whose duties are to be clearly defined.

### THE JAPANESE RED CROSS CONTINGENT: ARRIVAL IN LONDON.

The Anglo-Japanese Alliance, or, more broadly, the interdependence of East and West, which has received so many strange illustrations during the war, was again cordially marked by the arrival on Friday last week in this country of the Japanese Red Cross Society's contingent. Having travelled in the White Star liner *Megantic*, in response to the acceptation by the War Office of the Japanese Red Cross Society's offer of aid, the contingent consists of two surgeons, a secretary, two "chief nurses," twenty nurses, and an interpreter. The head of the party is Dr. Suzeki, who served before Port Arthur in the Russo-Japanese War. Dr. Oshima, another member, is a graduate of the Imperial University of Japan, and was actually studying medicine in Germany when war was declared. He managed to escape to London, and went on to Japan, which he left again with his present colleagues on December 19. Mr. N. Otsuka was professor at the Disciples' Bible College, Tokyo, and most of the party, including the nurses, have left their civil occupations, including in several cases their husbands or wives. The nurses, of whom Yama Moto is the chief, with Kiyookawho as her second-in-command, belong to the Red Cross League of Japan, which, founded some thirty years ago, binds its members, wherever they are, in case of emergency to respond to any call that may be made on them, and to go to wherever they may be sent during the period of three years' training and fifteen years' service in the Reserve. The party, which was received by Surgeon-General Sir Benjamin Franklin, K.C.I.E., on behalf of the British Red Cross Society, and a representative of the Japanese Embassy, will remain a week in London before proceeding to Netley. Mr. H. Bonar, formerly Consul-General at Seoul, has been attached to the party by the War Office.

### ROYAL VISIT TO THE EMPIRE HOSPITAL.

The King and Queen paid an informal visit to the Empire Hospital, Vincent Square, Westminster, on Tuesday afternoon last. During the visit, which extended over one hour, they chatted with each of the wounded officers accommodated in the building, and were conducted over the hospital. Some time was spent in the theatre block, and a visit also was paid to a little girl of three, occupying a room on one of the private floors, who was to be operated upon that evening. The King and Queen, on leaving, expressed their approval of the arrangements and equipment of the hospital.

### THE BURNLEY HOSPITAL COMPETITION.

An important step has been taken in the proposed extension of the Victoria Hospital for Burnley and District with the adjudication of Mr. R. Percy Adams, F.R.I.B.A., upon the plans sent in. Acting as assessor upon the plans submitted by the selected candidates Mr. Adams has now made his award, and the names of the architects of the four premiated plans in order of merit are as follows: First, Mr. William A. Pite, London; second, Messrs. Hitchon and Pickup, of Burnley, premium of £75; third, Messrs. Taylor and Simister, of Oldham, premium of £50; and fourth, Mr. T. H. Vowles, of Burnley, premium of £25. Ten sets of plans were sent in altogether, and among the competing architects was Mr. Saxon Snell, the remainder representing the profession in London, Manchester, Wolverhampton, and Burnley. It is thus apparent that a wide selection has been achieved by the competition, and with so experienced an assessor, provided that the advantage of having a hospital administrator's advice and counsel be fully made use of, the extension ought to be of first-rate quality in design, both architecturally and administratively.

### HERTFORD COUNTY HOSPITAL TAKES OUR ADVICE.

We are glad to see that our arguments in The Hospital of January 16, for a vigorous policy on behalf of Hertford County Hospital in the matter of raising funds, have been followed by an attempt to put Admiral Hastings' and Mr. Clinton Baker's views in its favour into successful action. That we echoed, like these gentlemen, the opinion of the best minds in the county is seen by the fact that the *Hertfordshire Mercury* has come forward and instituted a shilling fund, which it has prefaced with the following statement: "The capital account is the mainstay of the institution and it is false economy to weaken it. Because we feel that a mistake had been made," proceeds the journal, "that the public were largely unaware of the very serious crisis in the affairs of the hospital . . . we wrote to the governors offering to start and organise a shilling fund for the benefit of the hospital, and with a view to clearing off the deficit." As the journal also points out, following the lead we gave, "the sale of stock will not assist the reopening of the children's ward," and a definite objective of this kind is required to focus the public spirit of the county. To do this is to begin well, but the shilling fund must not be the end of the efforts. As a matter of fact, the hospitals to-day are in a unique position to secure prompt attention to their needs, since, notwithstanding the calls on the public purse, their work for the wounded, being a solid and generous contribution to the demands of the crisis, is the best possible advertisement for which they could wish. We therefore hope that Admiral Hastings and Mr. Clinton Baker will back up this statesmanlike effort by a definite campaign pointing out the part which the hospitals are playing, and definitely using this as a lever to consolidate that public interest in their welfare which the reception of the wounded has already awakened. We are glad to see that the clergy have been invited to come forward and co-operate in ear-marking an offertory on the hospital's behalf, and success will come if the newspaper, the pulpit, and, not least, the active spirits on the committee all unite to present the hospital's case and to instruct its supporters in the needs which the present situation has created, by meetings, lectures with possibly lantern slides, as well as collections. All experience proves that a collecting-box will remain empty unless arguments and interest are presented with it, for the latter alone can be sustained if the public are properly kept informed of the work being done and the problems to be faced.

### THE EXTENT OF WAR HOSPITAL ACCOMMODATION.

While we hear so much about the Red Cross hospitals and the amount of work for the wounded which is being done by the voluntary hospitals, in the strict sense, it is interesting to realise the extent of the total accommodation which the War Office has at its command. According to the past weekly report of the British Red Cross Society the number of hospitals accepted by the War Office up to date is no fewer than 686, and the number of beds 19,159. These figures form a record which will become interesting as a matter of history when in peaceful times we come to wonder how the urgent and extensive demand for additional hospital accommodation for the wounded was met. An analysis of these figures would reveal the respective parts played by improvised and permanent hospitals. It is a wonderful total, at any rate, and proves how capable the voluntary system is of responding to an emergency.

### HOSPITALS FOR POSTMEN.

The committee of the Post Office Relief Fund have opened home hospitals for the special accommodation of members of the Post Office Department serving with the Navy or Army who are invalided or otherwise incapacitated. A convalescent home has also been opened at Littlestone-on-Sea, in a house lent by Mrs. H. N. Gladstone for the purpose. The home hospitals at present consist of two converted private houses, one in Kensington Palace Gardens, and one in Ennismore Gardens, which have been placed at the disposal of the committee. It may be asked why the committee of the Post Office Relief Fund should have opened special hospital accommodation for incapacitated members of the Post Office Department, unless what is really convalescent treatment is being given under the more technical title. Presumably this is the case, since the wounded, as such, generally speaking, are being provided for in hospitals irrespective of the work in which they were engaged.

### THE CONDITION OF CONVALESCENT SOLDIERS.

As soon as the war broke out The Hospital devoted space to recording the large number of offers of private houses for the accommodation of sick and also of convalescent soldiers. The response was immediate, and it seemed that every county was dotted with private seats placed at the disposal of the Government as convalescent homes. It is now reported, however, that the supply of private accommodation has proved insufficient, and that while many soldiers when leaving hospital to recruit spend their convalescent period in charming surroundings it is yet a matter of luck what sort of reception the convalescent soldier meets with. The delightful conditions have been abundantly recorded in the illustrated newspapers; the reverse and less pleasing state of affairs is now reported in the *Times*. Crediton, the Devonshire country town a few miles from Exeter, is quoted as an instance in which resentment has been aroused at the accommodation provided for convalescent soldiers. There, it appears, some 230 wounded men from the county regiment, collected from hospitals all over the country, have been sent to convalesce, being forwarded to Crediton from the regimental depôt at Exeter. The convalescents are now housed in "a number of buildings which had been hurriedly fitted up for the accommodation of recruits." Public halls, clubs, cottages, which happened to be empty when the military authorities wanted rooms of any kind, are all used. The principal furniture consists of beds, made of straw, which are supported on planks raised by wood blocks a few inches above the ground. In fact, the whole accommodation is compared to that provided in the huts in the military camps. The authorities are reported to state that the men are contented and well, and that conditions will be shortly improved by the acquisition of a former boot factory, which can be converted into barracks. As the payment for billeted men amounts only to about 2s. a day there is a difficulty here. If the facts be indeed as reported, and if local resentment really has been aroused, the obvious remedy is the provision of further privately offered accommodation, which in the circumstances of a "stranded" regiment in the county whose name it bears should, we are convinced, immediately be responded to. A similar policy should be pursued in every county in which there is a shortage of convalescent accommodation.

### A DOCTOR SUES A COLLEAGUE FOR FEES.

A recent case—in Wales, we may note—saw the unusual event of one medical man suing another to recover fees for an operation. The plaintiff, Dr. Walton, of Shotton, Flintshire, stated that he attended two cases of tracheotomy, in which operations were performed on children at the request of the defendant, whom he had charged two guineas in each case. The defendant had paid the first, but not the second. The defendant, Dr. Whittome, of Connah's Quay, stated that while he had been paid by the parents in the first case, he had received nothing in respect of the operation performed on the second child. The judge remarked that he did not see any defence in law, since one doctor who engaged the services of another was liable for the fee. He gave judgment for the plaintiff with the usual costs. We agree with him, however, in thinking that it was a great pity that the case could not have been settled outside the Court. The rareness of such cases is sufficient to show that means can be found for doing so.

### THE WAR AND HOSPITAL ARCHITECTURE.

No one can now doubt, after six months of war, that the improvised hospital has come very largely to stay, although there have been instances in which the experiment of sending soldiers to them has been abandoned, always provided that better accommodation, in the hospital sense, was available. In spite, then, of a few miscarried schemes and endeavours, the authorities generally have been insistent more than once on the success which has attended the emergency hospital. Only this week, to give the latest instance, we find Sir Frederick Treves writing in the *Times* of the hospital work at Boulogne as follows: "At the back of the Maritime Station the goods sheds have been converted into a military hospital of the type known as stationary. They serve to show, better perhaps than anything in Boulogne, what the Army Medical Service of this country can accomplish. Major Norrington, R.A.M.C., the officer in charge, has changed these dismal and uninhabitable buildings into a series of bright wards, spotlessly clean, well lighted, well warmed, and well ventilated. This hospital is perfectly equipped, down to a special ward for pneumonia cases. . . There are two operating theatres in the hospital, both up to date in every point." There could be no higher praise than this. But we cannot stop thinking when we have congratulated ourselves on our magnificent Army Medical Service. We are forced to ask the question: What light does the success of this hospital, improvised out of goods sheds, throw upon the palatial and costly buildings that have been erected at almost every hospital extension of recent years? If the praise is true—as who can doubt?—the palatial buildings must have been unnecessary, and elaborate hospital architecture, as those who believe in the deductions to be drawn from modern aseptic treatment have sometimes averred, must be very largely money wasted. It is a question worth considering. You cannot have it both ways. If the work done in the improvised goods sheds is as good in results as that in a building costing several hundred pounds a bed, the expensive building is an extravagance. If the carefully designed and costly hospital is necessary to the best results, then the success of our emergency hospitals has been the success of a splendid makeshift only. Here is a point for architects and committeemen to consider? What are their opinions?

### THE INSURANCE ACT IN LONDON.

A Report presented to the London Insurance Committee at its meeting on January 28 showed that the number of medical practitioners on the panel at the end of the year was 1,569, of whom 1,463 were in general practice and 106 in institutions. This is a small increase on the number on the panel at the end of June, from which it would appear that insured persons in London are not suffering from a shortage of doctors in consequence of the war. The places vacated by panel doctors who are absent from home in connection with the war have in fact been filled by other doctors, so that as far as insured persons are concerned no inconvenience is being caused. The number of chemists on the panel at the end of the year was 819, which is three more than at the end of June. Panel chemists, by the way, are faced by an unhappy prospect, for the Report of the Finance Sub-Committee states that "Our attention has again been drawn to the large demand which has been made upon the Drug Fund for the medical year 1914. From the figures which have been submitted to us for consideration it would appear that the amount available for payment to persons supplying drugs and appliances during the medical year 1914 will be about 75 per cent. of the total amount of the accounts as furnished to the Committee, and that the estimated deficit will be approximately £46,000." This is a very serious matter for the chemists. A statement submitted by the Sanatorium Benefit Sub-Committee shows that the number of insured persons suffering from tuberculosis who were receiving treatment on December 31, 1914, was 2,714, of whom 1,931 were men and 783 women; the number being treated in hospitals was 189 and in sanatoria 500; the number undergoing dispensary treatment was 790, and the number under treatment in their own homes 1,235, or very nearly one-half, which is probably too large a proportion to produce satisfactory results from the treatment.

### IMPROVEMENTS AT COLNEY HATCH MENTAL HOSPITAL.

The London Mental Hospitals Committee are about to proceed with a further stage in the improvement of Colney Hatch Mental Hospital, this being the alteration of "D" Ward, which is said to call for attention more urgently than the remainder. This ward at present contains dormitory space for thirty-nine patients and four attendants. It is proposed to abolish nine of the single rooms and two attendants' rooms, and by the addition of their area to increase the dormitory accommodation of the ward to forty-nine beds. The altered ward and four attendants' rooms will then accommodate ten more patients than at present. Additional day-room space will be provided by the inclusion of a part of the area of certain old workshops adjoining the ward and an additional sanitary annexe, clothes stores and a boot room are also to be provided. The lighting and ventilation will be improved by the provision of additional windows and the replacement of the existing iron window-frames by new and enlarged wooden sashes. The roof of the low-level corridor will be lowered to provide cross-ventilation. The work is to be carried out by direct labour, under the supervision of the mental hospitals' engineer, and the County Council has approved an expenditure of £2,540 for the work.

### THIS WEEK'S DRUG MARKET.

The demand for drugs is now at least as large, if not larger, than it was at the corresponding season last year. In some cases, as, for instance, morphine, codeine, chloroform, iodides, and mercurials, the output is much greater than usual owing to the requirements of the Medical Services with the Armies. Salicylic acid and the salicylates continue to tend upwards in price as a result of shortage of supplies, and still higher quotations are by no means improbable, notwithstanding that present values are from five to six times higher than those quoted before the war; economy in prescribing drugs of this series seems to be indicated. Opium is practically unchanged, and the prices of morphine and codeine are firmly maintained. Acetanilide is again dearer, and the quotation for phenacetin has been raised. Quicksilver again has a rising tendency in price, but so far the values of corrosive sublimate and calomel have not been further advanced. The price of santonin is not quite so firmly maintained, but extreme rates will no doubt have to be paid for a long time.

